# Stress Management as an Effective Complementary Therapeutic Strategy for Weight Loss in Children and Adolescents with Obesity: A Systematic Review of Randomized Controlled Trials

**DOI:** 10.3390/children8080670

**Published:** 2021-07-31

**Authors:** George Paltoglou, George P. Chrousos, Flora Bacopoulou

**Affiliations:** 1 University Research Institute of Maternal and Child Health & Precision Medicine and UNESCO Chair in Adolescent Health Care, National and Kapodistrian University of Athens, 11527 Athens, Greece; gpaltoglou@med.uoa.gr (G.P.); chrousos@gmail.com (G.P.C.); 2 Division of Endocrinology, Metabolism and Diabetes, First Department of Pediatrics, School of Medicine, National and Kapodistrian University of Athens, Aghia Sophia Children’s Hospital, 11527 Athens, Greece; 3 Center for Adolescent Medicine and UNESCO Chair in Adolescent Health Care, First Department of Pediatrics, School of Medicine, National and Kapodistrian University of Athens, Aghia Sophia Children’s Hospital, 11527 Athens, Greece

**Keywords:** stress management, MBSR, mindfulness, obesity, children, adolescents, eating intervention, lifestyle

## Abstract

Lifestyle intervention programs, including mindfulness and stress management/emotional control training techniques have been infrequently studied in children. The aim of this systematic review was to assess whether implementing stress management/emotional control training strategies in the context of a body weight loss program in children and adolescents is associated with improved body weight outcome in this age group. A systematic literature search was conducted to identify relevant studies published before 31 December 2020 in the following databases: Medline (PubMed), Scopus, and Cochrane Central Registry of Controlled Trials. Only randomized clinical trials (RCTs) on mindfulness or stress management in children and adolescents with obesity were included in this systematic review. Six RCTs fulfilled the study inclusion criteria and included intervention (112 subjects) and control (137 subjects) groups. The interventions used were Mindfulness-Based Stress Reduction therapy for 8 weeks (three studies), a mindfulness-based group program for adolescents (one study), and Mindful Eating Intervention for 6 weeks (one study) and 10 weeks (one study). The intervention group demonstrated reduced adiposity markers as compared to controls in four of the six included studies. The presented studies support the hypothesis that a structured, mindfulness-based intervention program may lead to a decrease in the biomarkers of obesity.

## 1. Introduction

Despite notable progress in the understanding of overweight/obesity as a pathophysiologic process, which in many individuals commences in early childhood, and of the basic science behind maintaining an appropriate amount of body fat, its management remains a challenge for both the affected person and healthcare providers [1,2]. At the same time, treating overweight/obese children and/or adolescents is difficult and requires changes in diet, physical activity, sleep, and environment [3]. This is underpinned by many studies that showcase an increase in obesity prevalence throughout childhood worldwide, while also stressing the importance of efforts in maximizing preventive intervention opportunities prior to the onset of this process [4,5]. Of note, body weight maintenance in a growing child or adolescent is as effective as weight loss in an adult, and lifestyle changes—although evidently not as beneficial as for adults—are recommended [3].

Lifestyle interventions that have been proposed for children and adolescents can be associated with individually perceived stress [6]. At the same time, obesogenic behaviors, such as consumption of calorie-dense, “nutrient-poor” foods, sugar- or artificially-sweetened beverages, lack of physical activity, unhealthy sleep patterns, and excessive screen time, are behaviors that have been associated with increased stress system activation [7,8,9,10,11,12]. It has also been reported that during times of increased perceived stress, people show less inhibited eating behaviors [6]. Obesity and its associated illnesses have also been related to chronic psychosocial stress and its contributing factors (poor interpersonal relationships, job or unemployment stress, poor self-esteem, low socioeconomic status, etc.) [13].

Interestingly, there is interindividual variation, genetically determined, in the biological response to stress, which may lead to higher vulnerability to mental or physical stressors [14]. Early life stress has been associated with multiple pathologic somatic and behavioral pathways in children, which may increase the risk for later obesity [15,16]. Longitudinal studies and meta-analyses have provided evidence of a significant association between attention deficit hyperactivity disorder (ADHD) symptoms and obesity, regardless of possible confounding factors, incriminating abnormal eating patterns, sedentary lifestyle, and possibly common genetic alterations [17]. The chronic distress characteristics of stressed individuals contribute to the development of obesity via increased emotional “comfort” eating, lack of sleep, impulsive behaviors, selection of specific calorie-dense foods, etc. [18].

Apart from poor diet- and sedentary lifestyle–oriented behavioral treatment, strategies targeting psychosocial factors, such as emotional distress, have been proposed [19]. Among the behavior change techniques that have been tried for obesity, there is growing evidence of the benefit of implementing stress management/emotional control training [3]. Mindfulness, as defined by Kabbat-Zin, is “the awareness that emerges through paying attention to the present moment nonjudgmentally, as experience is unfolding moment by moment”. This method has to do with particular qualities of attention and awareness that can be cultivated and developed through meditation [20]. To better help people with chronic health problems and those suffering from chronic psychosocial stress adapt to and tolerate life challenges, Mindfulness-Based Stress Reduction (MBSR) was developed [20,21]. MBSR in weight management is a psycho-educational intervention during which participants train in mindfulness-based practices and learn about proper nutrition, rest, and exercise, as well as the role played by thoughts and emotions in physical and emotional health [22]. Mindfulness can thus be defined as “the learned ability to be open, accepting, and present in the moment” and has been posited to be an effective intervention for the treatment of both psychological and physical symptoms [23,24]. In the context of body weight management, mindfulness has gained attention as an avenue for the treatment of obesity through modification of eating, exercise, and sleep, and as an adjuvant/complementary strategy [25].

The aim of the present systematic review was to assess whether implementing stress management/emotional control training strategies in the context of a body weight loss program in children and adolescents is associated with improved weight outcome in this age group. Secondary outcomes of the studies included were also assessed.

## 2. Materials and Methods

This study was conducted in accordance with the recommendations of the Preferred Reporting Items for Systematic Reviews and Meta-analyses (PRISMA) statement. The study protocol included the following steps: (i) original systematic literature search; (ii) selection of appropriate studies to be included. Only randomized controlled trials (RCTs) were included in this systematic review.

A systematic literature search was conducted to identify RCTs on mindfulness or stress management in children and adolescents with obesity. The search was conducted for studies published before 31 December 2020 in the following databases: Medline (PubMed), Scopus, and Cochrane Central Registry of Controlled Trials. Two groups of keywords proposed by a group of experts with relevant methodological and clinical expertise were used. The Medical Subject Headings (MeSH) database was used for identification of synonyms. These two groups were combined by the Boolean “AND”, and the terms utilized within these search categories were combined by the Boolean “OR”. The full search strategy used for Pubmed was as follows: (“mindfulness” [All Fields] OR “stress management” [All Fields]) AND (“child*” [All Fields] OR “adolescen*” [All Fields]) AND “obes*” [All Fields] (Filters applied: Clinical Study). This was adapted appropriately for the rest of the databases. Reference lists of selected articles were used to find additional studies that were not retrieved in the initial search. Risk of bias assessment was performed independently by the researchers by applying the JADAD scale [26] to each individual study, as reported in Table 1 [27,28,29,30,31,32]. No study was blinded for the intervention, and only the study by Kumar et al. [32] was blinded for the statistician and the assessor.

## 3. Results

**Figure 1:**
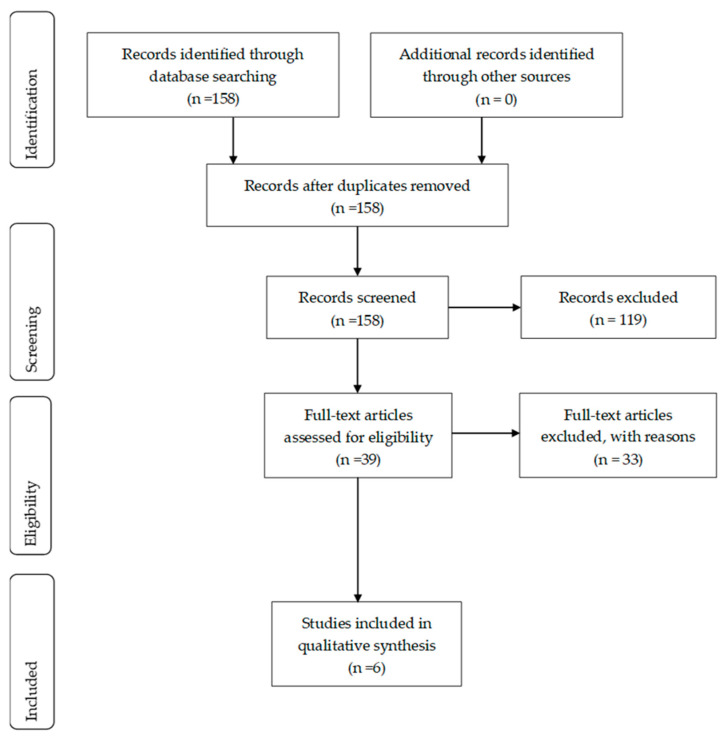
Flow chart of study selection.

Following the original systematic literature search, duplicates were excluded. The remaining studies were evaluated by two independent researchers according to the inclusion and exclusion “study eligibility criteria”. From the original 158 retrieved studies, 119 were listed as reviews and excluded. From the remaining 39 studies, following full text analysis, six RCTs [27,28,29,30,31,32] were included in this systematic review, and the remaining 33 were excluded. The study selection process is presented in Figure 1.

All studies included an intervention group (112 subjects) and a control group (137 subjects). The interventions used were Mindfulness-Based Stress Reduction (MBSR) therapy for 8 weeks (three studies), a mindfulness-based group program for adolescents (one study), and Mindful Eating Intervention (MEI) for 6 weeks (one study) and 10 weeks (one study). The intervention groups showed reduced adiposity markers, as compared to controls, in four of the six included studies. The characteristics of the included studies are shown in Table 2.

## 4. Discussion

It was shown that an 8 week stress management intervention program that includes progressive muscle relaxation, diaphragmatic breathing, guided imagery, and cognitive restructuring significantly reduces BMI or waist-to-hip ratio, without a simultaneous BMI z-score reduction, in two studies of Greek obese children and adolescents [27,28]. Higher BMI during childhood is associated with an increased risk of coronary heart disease in adulthood, while it is noted that many obese adolescents already have cardiometabolic co-morbidities [33,34]. This underlies the important concept that even a mild reduction in body weight before the onset of puberty can significantly reduce the risk for cardiovascular and metabolic disease later in life [34]. It also makes paramount every effort to improve the efficacy of body weight management programs in childhood and adolescence.

Implementing a complementary mindfulness treatment strategy might improve central body fat deposition [28]. Central body fat deposition in adults is a risk factor for carbohydrate intolerance, type 2 diabetes mellitus, dyslipidemia, systemic arterial hypertension, and coronary artery disease, an association that holds true in children and adolescents [35,36]. Adipose tissue is an active endocrine organ secreting bioactive peptides, known as adipokines or adipocytokines, acting as efferent signals with both local (autocrine/paracrine) and systemic (endocrine) effects [36,37]. At the same time, adipose tissue expresses numerous hormone receptors that allow it to respond to afferent signals from traditional hormone systems as well as from the central nervous system (CNS) [37]. The interplay of all of these hormonal and neural signals in obesity is potentially a causative link—together with the obesity-associated smoldering systemic inflammation (para-inflammation) and oxidative stress—connecting obesity with its devastating sequelae [36]. It has been shown that upper-body and visceral fat decrease to pre–weight-gain levels more rapidly than lower-body fat during weight loss [38]. This preferential time- and body region–limited loss might reflect differences in triglyceride storage capacity or lipolysis rates between lower- and upper-body adipocytes [38,39,40].

In addition, in the study by Stavrou et al., the stress management methods applied resulted in a significant decrease in depressive and anxiety symptoms and led to a reduction of the internalizing and externalizing behaviors in children and adolescents participating in the intervention group [27]. Depressive and anxiety symptoms have been associated with both developing and long-standing obesity, particularly when considering the distressing sequelae of obesity, which is detrimental to quality of life [41,42]. Of particular importance is the study by Emmanouil et al., in which an improvement in daily habits and school performance was reported, suggesting that stress management has a favorable effect on the constellation of obesity, anxiety/depression, and educational attainment [28]. This might be the mechanistic parameter influencing the potential of maintaining body weight loss when using a mindfulness-based intervention [22].

Functional imaging studies have shown that MBSR-trained, compared to -untrained subjects, have increased brain connectivity during a focused attention instruction and show greater differentiation between attended and unattended sensory networks [43]. There are also reports indicating that mindfulness might attenuate the increased functional connectivity of the amygdala observed during perceived stress [44]. This is of particular importance as the central nucleus of the amygdala stimulates the stress system and forms a mutually reinforcing positive feedback loop [45]. A recent study of mindfulness training in people who have lost weight and intend on maintaining it showed increased functional connectivity in a neural circuit involved in emotion regulation and depression symptoms [46].

Still, when adolescents at risk, as defined by either a BMI exceeding the 70th percentile or having two parents with obesity, received 6 weeks of stress management intervention, as compared to a control group that received only general health education, equivocal results were seen [29]. As noted by the authors, this finding highlights the importance of a more restrictive selection of participants to better assess the effect of stress management on the BMI of overweight and obese adolescents [29]. Clinical research eligibility criteria play an essential role in clinical and translational research, particularly in children where optimal measures of adiposity comprise a separate field of research [47]. In addition, it has been posited that it is critical to consider individual variability in training responsiveness in the application of mindfulness training, both in clinical settings and in the public domain, while there are indications that mindfulness may work better in specific subpopulations [22,48]. There is also evidence supporting the assumption that the extent to which individuals benefit from mindfulness training might reflect individual differences in the innate ability to focus attention on present-moment experiences and to maintain present-moment awareness with a non-judgmental and nonreactive attitude (trait mindfulness) [48].

An alternative approach termed “Mindful Eating Intervention” was shown to be effective in Latino adolescent girls [30]. When the sample received 6 weeks of MEI, BMI decreased as compared to the matched control group [30]. Mindful eating adds cognitive elements and posits to make an individual able to have a “non-judgmental awareness of [one’s] physical and emotional sensations while eating or in a food-related environment” [25]. Cognitive behavioral therapy (CBT) is a theoretically based treatment approach that emphasizes the process of changing habits and attitudes that maintain psychological disorders and could be an appropriate treatment approach to obesity [49]. More specifically, CBT recognizes that cognition, feelings, and behaviors are interrelated and utilizes techniques involving motivational enhancement, goal setting, problem solving, and knowledge/skill acquisition that can facilitate sustainable behavior changes [50]. Mindfulness-based cognitive therapy (MBCT) is an adaptation that incorporates elements of CBT with mindfulness-based stress reduction, aiming to teach those at high risk of depressive relapse to stay well through learned skills [21,51]. Mindfulness with or without specific training in mindful eating may help people better control portion sizes and choose less calorie-dense foods while improving appreciation for food [52,53]. There is evidence that the improved appreciation (eating enjoyment—“joyful eating”) of mindful eating strategies focusing on the sensory properties of food result in lower energy intake from unhealthy foods [54].

An 8-week parent–child dyad stress management program employed to prevent childhood obesity was shown to positively affect child BMI percentile after accounting for changes due to positive and negative parenting [31]. The observed positive changes could potentially be attributed to the improved structured parental involvement (improvement in positive parenting) as well as improved eating and physical activity choices for the children [31]. Family-based MEI was implemented in an adolescent group, in whom, despite no dropouts, no difference in BMI was observed between the intervention and control groups [32]. It has been reported that mindful eating as a construct may have the potential to mediate the relation between individual parenting style and child eating behavior (including emotional overeating) [55]. Interestingly, novel, integrated approaches aim to include both children/adolescents and their parents (mindful parenting) and focus on eating behavior in the family context [56].

In summary, there is supportive evidence in the literature that a structured 6- or 8-week stress management, mindfulness-based, therapeutic program might lead to a decrease in the biomarkers of obesity, including BMI and/or waist-to-hip ratio. These mindfulness-based therapeutic strategies focus on the child and adolescent mainly through the parent–child relationship. Not to be overlooked, the low dropout rate reported among the participants in the reviewed studies suggests that stress management improves adherence to a healthy lifestyle if used as a complementary therapeutic method; this is a promising focus of future research. The main limitation of this systematic review is that only qualitative analysis of the included studies could be performed, due to the small number of published studies available. Although the studies were randomized, it was inherently impossible for the intervention to be blinded.

In conclusion, mindfulness-based stress management programs may be a vehicle to develop awareness of and compassion toward one’s self and a way to improve parental, childhood and adolescent emotional well-being and control of stress, thus improving the quality and quantity of food intake towards a healthier pattern. Parents, in particular, are a major determinant of the obesogenic environment in which children grow and develop. Furthermore, the mechanisms underlying the effectiveness of mindfulness-based interventions are a budding field of research in stress management to ameliorate stress-associated morbidities. The inclusion of educational components could help with the management of different situations in day-to-day life.

## Figures and Tables

**Table 1:** JADAD 5-point scale for risk assessment bias. The authors independently marked each individual study. Conflicting results were resolved following discussion.

Article	Randomization	Blinding	Withdrawals and Dropouts	Total Score
Stavrou, S. et al. *J. Mol. Biochem.* 2016 [27]	2	0	1	3
Emmanouil, C.C. et al. *Hormones* 2018 [28]	2	0	1	3
Shomaker, L.B. et al. *Appetite* 2019 [29]	2	0	1	3
Daly, P. et al. *Comp. Ther. Med.* 2016 [30]	2	0	1	3
Jastreboff, A.M. et al. *J. Pediatr.* 2018 [31]	2	0	1	3
Kumar, S. et al. *Child* 2018 [32]	2	1	1	4

**Table 2:** Characteristics of included randomized controlled trials.

Reference	Participants’ Age, N	Intervention	Outcome
Stavrou et al. *J. Mol. Biochem.* 2016 [27]	11.15 ± 1.48 years Control: 26 Intervention: 23	Intervention and control groups: nutritional and physical activity counseling. Intervention: 8 weeks of Mindfulness-Based Stress Reduction therapy.	Intervention group: BMI reduction
Emmanouil et al. *Hormones* 2018 [28]	8–17 years Control: 20 Intervention: 16	Intervention and control groups: nutritional and physical activity counseling. Intervention: 8 weeks of Mindfulness-Based Stress Reduction therapy.	Intervention: reduction of waist-to-hip ratio
Shomaker et al. *Appetite* 2019 [29]	12–17 years Control: 25 Intervention: 29	Control group: 6 weeks health education group program for adolescents Intervention group: 6 weeks mindfulness-based group program for adolescents.	No difference in adiposity markers
Daly et al. *Comp. Ther. Med.* 2016 [30]	14–17 years Control: 23 Intervention: 14	Control group: 6 weeks health education group program for adolescents Intervention group: 6 weeks Mindful Eating Intervention (MEI).	Intervention group: lower BMI
Jastreboff et al. *J. Pediatr.* 2018 [31]	44.8 ± 13.8 months 42 parent–child dyads Control: 22 Intervention: 20	Intervention and control groups: nutritional and physical activity counseling. Intervention group: 8 weeks of Mindfulness-Based Stress Reduction therapy.	Control group: increased BMI centiles
Kumar et al. *Children (Basel)* 2018 [32]	14.5–17.9 years 22 parent–adolescent dyads Control: 11 Intervention: 11	Intervention and control groups: nutritional and physical activity counseling. Intervention group: 10 weeks Mindful Eating Intervention (MEI).	No difference in BMI

## Data Availability

Not applicable.
